# Genetic evaluation of heat tolerance in Holstein cows in Japan

**DOI:** 10.1111/asj.13437

**Published:** 2020-08-05

**Authors:** Koichi Hagiya, Yamato Atagi, Takefumi Osawa, Takeshi Yamazaki

**Affiliations:** ^1^ Obihiro University of Agriculture and Veterinary Medicine Obihiro Japan; ^2^ Graduate School of Agricultural and Life Science University of Tokyo Tokyo Japan; ^3^ National Livestock Breeding Center Nishigo Japan; ^4^ NARO Hokkaido Agricultural Research Center Sapporo Japan

**Keywords:** dairy cow, heat stress, heat tolerance, Holstein, random regression model

## Abstract

We used test‐day records and daily records from provincial weather stations in Japan to evaluate heat tolerance (HT) in Holstein cows according to a random regression test‐day model. Data were a total of 1,641,952 test‐day records for heritability estimates and 17,245,694 test‐day records for genetic evaluation of HT by using milk yield and somatic cell score (SCS) in Holstein cows that had calved for the first time in 2000 through 2015. Temperature–humidity index (THI) values were estimated by using average daily temperature and average daily relative humidity records from 60 provincial Japanese weather stations. The model contained herd–test‐day, with lactation curves on days in milk within month–age group as a fixed effect. General additive genetic effect and HT of additive genetic effect were included as random effects. The threshold value of THI was set to 60. For milk yield, estimated mean heritabilities were lower during heat stress (THI = 78; 0.20 and 0.28) than when below the heat stress threshold (THI ≤ 60; 0.26 and 0.31). For SCS, heritability estimates (range 0.08–0.10) were similar under all heat stress conditions. Genetic trends of HT indicated that EBVs of HT are changing in an undesirable direction.

**Implications**

Heat stress in Holstein cattle is a growing concern in Japan because of its negative effects on dairy cows and associated economic losses in the dairy industry. To improve cow’ heat tolerance, we investigated the heritabilities of heat tolerance based on milk yield and cow health. Correlations between genetic performances of heat tolerance estimated by using milk yield and those based on a cow health indicator were close to zero. Therefore, to improve heat tolerance in dairy cattle, we recommend selecting animals that are concurrently superior according to heat tolerance based on milk yield and as well as cow health.

## 1 INTRODUCTION

The negative effects of heat stress (HS) in Holstein cows are well documented (e.g., Hayes, Carrick, Bowman, & Goddard, 2003; West, 2003) and generally are estimated by using the temperature–humidity index (THI). Effects of HS, which occurs when the THI exceeds a threshold value, lead to economic losses (St‐Pierre, Cobanov, & Schnitkey, 2003). In recent studies, threshold THIs were between 60 and 65 (Hagiya et al., 2019; Nguyen et al., 2017). Test‐day milk yield decreases as THI increases above the threshold value (Ravagnolo & Misztal, 2000); similarly, test‐day somatic cell count (SCC) in milk increases with increasing THI (Hammami, Bormann, M’hamdi, Montalda, & Gengler, 2013; Lambertz, Sanker, & Gauly, 2014).

Weather‐wise, Japan is quite humid, especially in summer, and Holstein cows in southern areas experience severe HS during this season (Hagiya et al., 2017). Several studies have reported various effects of HS in Holstein cows in Japan (Atagi et al., 2018, 2019; Hagiya et al., 2019; Nagamine & Sasaki, 2008). According to a simulation study of global warming that estimated temperature according to various RCP (Representative Concentration Pathway) scenarios for CO_2_ conditions, average annual temperatures will increase by 0.3°C to 4.8°C in the 21st century (Ministry of the Environment, 2018). Therefore, in Japan, genetic improvement of heat tolerance (HT) is gaining interest in the dairy industry.

Ravagnolo and Misztal (2000) proposed a model to estimate additive genetic variances caused simultaneously by general production and HT through random regression on THI and containing repeatability of animal effects on test‐day milk yields. Bohmanova, Misztal, Tsuruta, Norman, and Lawlor (2008) applied their genetic evaluation methodology for HT to 56 million test‐day records from first‐parity Holstein cows in the United States. Aguilar, Misztal, and Tsuruta (2009) extended the model to evaluate HT by using milk, fat, and protein yields in a random regression test‐day model on days in milk (DIM). Bernabucci et al. (2014) suggested that HT selection should be included in selection objectives, owing to existing unfavorable relationships between HT and milk production. Carabaño, Bachagha, Romón, and Díaz (2014) evaluated HT by using complex models containing higher than one‐order Legendre polynomial regression on temperature hot and dry conditions in Spain. Nguyen et al. (2017) reported implementation of a breeding value for HT of dairy cattle in Australia and found that genetic trends declined over time. Atagi et al. (2018) reported genetic parameters of HT by using a random regression model on DIM and THI for milk, fat, protein, and somatic cell score (SCS) in southern Japan.

The aim of our current study was to estimate the genetic parameters of cows for HT, as indicated by changes in milk yield and SCS according to THI, by using a random regression model that applied information from daily weather records collected at provincial weather stations throughout Japan. In addition, we compared the estimated breeding values (EBVs) of HT between estimates based on milk and on SCS.

## 2 MATERIALS AND METHODS

### 2.1 Data

Data contained test‐day records of milk and SCC in Holstein cows that had calved for the first time in 2000 through 2015. Records were collected through the Dairy Herd Improvement Program and were provided by the Livestock Improvement Association of Japan (Tokyo, Japan). These data included records of test‐day milk yield and SCC for first‐lactation cows from all over Japan. SCCs were log‐transformed into SCSs by using the formula SCS = log_2_(SCC/100,000) + 3 (Ali & Shook, 1980).

Weather records from 60 provincial weather stations were obtained from the website of MeteoCrop DB (National Institute for Agro‐Environmental Science, NARO, 2017). THI values were estimated by using average daily temperature and average daily relative humidity, according to Hagiya et al. (2019). First, THI was estimated by using the following formula (NRC, 1971):

(1) $$\text{THI}=1.8\times t+32‐\left(0.55‐0.0055\times \text{rh}\right)\times \left(1.8\times t‐26\right),$$

where *t* is temperature in degrees Celsius and rh is relative humidity as a percentage.

Test‐day records were linked to data from each of the nearest weather stations of the 14 on Hokkaido, Japan's northernmost island, and from stations in the other 46 prefectures. Hagiya et al. (2019) reported that the lag in response to a heat effect was about 3 days for milk yield and 8 to 10 days for SCC under Japanese weather conditions. Therefore, we set the length of the lag in response on test day to represent heat effects as 3 and 8 days for milk yield and SCS, respectively. To estimate genetic parameters, 10 subsets of data were chosen randomly according to herd; each subset included more than 140,000 records and 40,000 animals (Table 1). Data for genetic evaluation contained 17,245,694 test‐day records from 2,018,402 cows. The pedigree file was provided by the Holstein Cattle Association of Japan (Tokyo, Japan) and comprised data on 2,987,536 animals, including cows with test‐day records and whose lineage could be traced back five generations.

**TABLE 1:** Number of records, mean, and standard deviation (*SD*) in each data subset

Subset	*N*	Milk		Somatic cell score	
		Mean	*SD*	Mean	*SD*
Parameter estimation					
0	142,315	27.0	6.2	2.31	1.66
1	164,046	27.1	6.4	2.39	1.65
2	159,092	26.8	6.4	2.40	1.65
3	185,925	26.5	6.6	2.41	1.67
4	172,369	26.8	6.2	2.37	1.61
5	176,599	26.9	6.5	2.36	1.63
6	148,193	26.7	6.4	2.35	1.64
7	164,694	27.1	6.5	2.34	1.66
8	180,694	26.8	6.5	2.41	1.66
9	148,025	27.0	6.2	2.33	1.64
Genetic evaluation					
	17,245,694	27.1	6.3	2.37	1.62

### 2.2 Model

Genetic and environmental (co)variances for milk yield and SCS were estimated by using a random regression animal model. The general additive genetic and general permanent environmental (PE) effects were modeled by using the Legendre polynomial function, with third‐order functions for milk yield and second‐order functions for SCS. We chose fixed effects by referring to the national genetic evaluation for production traits in Japan (Hagiya, 2019), as follows:

(2) $$\begin{array}{cc} y_{\mathrm{ijklt}}= & {\text{HTD}}_{i}+\sum_{l=0}^{5}{\text{AM}}_{\mathrm{jl}}w_{t}+\sum_{l=0}^{m}u_{\mathrm{kl}}v_{t}+uh_{k}f\left({\text{THI}}_{i}\right)+\\ & \sum_{l=0}^{m}{\text{pe}}_{\mathrm{kl}}v_{t}+peh_{k}f\left({\text{THI}}_{i}\right)+e_{\mathrm{ijklt}}, \end{array}$$

where $y_{\mathrm{ijklt}}$ = observation of test‐day milk or SCS; HTD*_i_* = fixed effect of herd × test‐day i; AM*_jl_* = fixed regression coefficients l for lactation curves of age × month j (15 age groups × 12 calendar months = 180 subclasses); $u_{\mathrm{kl}}$ = the general random additive genetic effect of animal k corresponding polynomial coefficients (covariates) l for lactation curve; $uh_{k}=$ the random additive genetic effect of HT of animal k corresponding linear regression coefficient (covariate) for HS in herd i; ${\text{pe}}_{\mathrm{kl}}$ = the general random PE effect of animal k corresponding to polynomial coefficients (covariates) l for lactation curve; $peh_{k}=$ the random PE effect of HT of animal k corresponding linear regression coefficient (covariate) for HS in herd i; $e_{\mathrm{ijklt}}=$ vectors of random residual effects; $w_{t}\prime =\left[\begin{array}{cccccc} \emptyset_{0}\left(t\right) & \emptyset_{1}\left(t\right) & \emptyset_{2}\left(t\right) & \emptyset_{3}\left(t\right) & \emptyset_{4}\left(t\right) & e^{‐0.05t} \end{array}\right]$, or the fourth‐order Legendre polynomial and Wilmink's function (Wilmink, 1987) at DIM t; $v_{t}\prime =\left[\begin{array}{ccc} \emptyset_{0}\left(t\right) & \cdots & \emptyset_{m}\left(t\right) \end{array}\right]$, or the mth‐order Legendre polynomial function at DIM t; and THI in herd × test‐day i as the function of HS.

$$f\left({\text{THI}}_{i}\right)=\left\{\begin{array}{c} 0\;\text{if}\;{\text{THI}}_{i}\leq \text{threshold}\\ \left({\text{THI}}_{i}‐\text{threshold}\right)\;\text{if}\;{\text{THI}}_{i}>\text{threshold} \end{array}\right).$$

The THI threshold value was set to 60 for milk yield and SCS, according to recent reports (e.g., Ammer, Lambertz, & Gauly, 2016; Hagiya et al., 2019; Hayes et al., 2003; Lambertz et al., 2014; Nguyen et al., 2017; Nguyen, Bowman, & Haile–Mariam M, Pryce JE and Hayes BJ, 2016). The (co)variance structure of random effects was

$$\text{var}\left[\begin{array}{c} u\\ p\\ e \end{array}\right]=\left[\begin{array}{ccc} \left[\begin{array}{cc} G & g\\ g\prime & \sigma_{\mathrm{uh}}^{2} \end{array}\right]⊗A & 0 & 0\\ 0 & P⊗I & 0\\ 0 & 0 & R \end{array}\right],$$

where *G* = (m + 1) × (m + 1) additive genetic (co)variance matrix for u; g= additive genetic covariance vector between u and uh;$\sigma_{\mathrm{uh}}^{2}=$ additive genetic variance for uh; ⊗= the Kronecker product; A= the additive genetic relationship for animals; P= (m + 2) × (m + 2) PE (co)variance matrix for pe and peh; I= the identity matrix for cows; and R= the residual variance.

We tested two models for random regression on DIM. Model 1 contained m= 0 (i.e., additive genetic variance and PE variance were constant throughout the lactation period), and Model 2 contained m= 3 for milk yield and m= 2 for SCS (i.e., random regression was adopted for variances changing by DIM). In the preliminary study, we tried third‐order Legendre polynomial functions for genetic and PE effects on SCS, but most subsets did not yield appropriate convergence.

Genetic correlations between HT on ${\text{THI}}_{i}$ and milk yield or SCS at DIM t were obtained by

$$r_{G}=\frac{v_{t}\prime g\times f\left({\text{THI}}_{i}\right)}{\sqrt{v_{t}\prime Gv_{t}\times f{\left({\text{THI}}_{i}\right)}^{2}\sigma_{\mathrm{uh}}^{2}}}.$$

Genetic parameters for the random regression models were estimated by using the AIREMLF90 program (Misztal et al., 2002), and EBVs from Model 1 and from Model 2 were estimated by using the MMEF program (T. Osawa, personal communication, 19 March 2020) with the preconditioned conjugate gradients method, which is used to estimate the national genetic evaluation in Japan. We compared the correlations between EBVs of THI from Model 1 with those from Model 2.

## 3 RESULTS

### 3.1 Summary statistics

**FIGURE 1:**
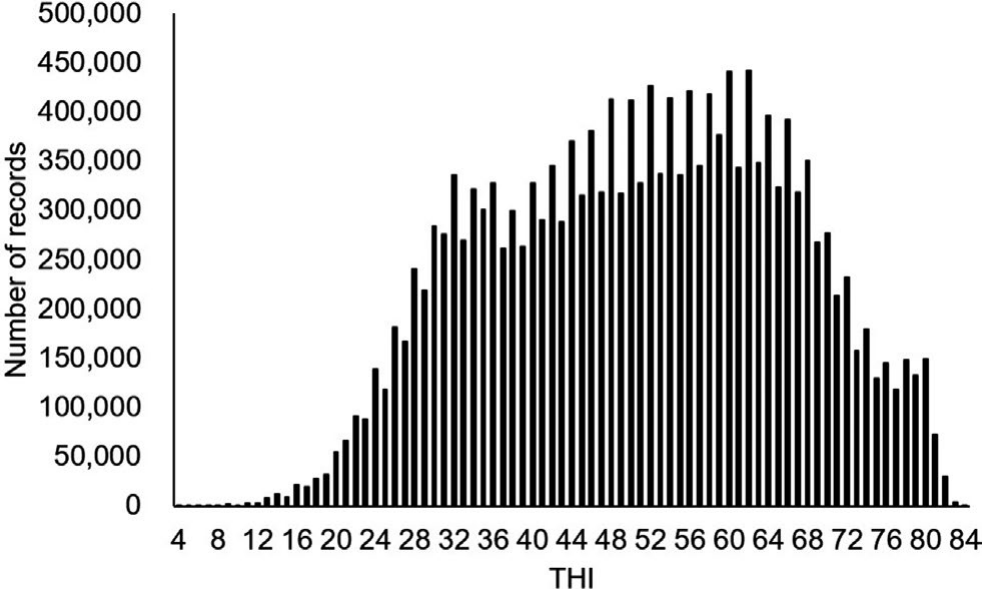
Frequency of the temperature–humidity index (THI) on test days

Means of milk yield and SCS are given in Table 1. Daily milk yield ranged from 26.5 to 27.1 kg and daily SCS from 2.31 to 2.41 (Table 1). THI ranged from 4 to 84 (Figure 1). The average THI was 50.6 and the mode was 62.

### 3.2 Changes in heritabilities on THI

Genetic parameter estimates of test‐day milk yield were obtained from all subsets for Model 1 and from 8 subsets in Model 2 (Table 2). Mean estimate of heritability at THI ≤ 60 was 0.26 in Model 1 and 0.31 in Model 2. Both models estimated lower heritabilities (0.20 and 0.28) under HS (THI = 78). For SCS, genetic parameters were estimated from all subsets for Model 1 and from 9 subsets for Model 2 (Table 3). Estimated heritability at THI ≤ 60 was 0.08 for Model 1 and 0.09 for Model 2. Heritability under HS (THI = 78) was 0.09 for Model 1 and 0.10 for Model 2. Heritability estimates for SCS were similar, regardless of THI.

**TABLE 2:** Means of estimated heritabilities from 6 DIM to 305 DIM for test‐day milk yield in each data subset

Subset	Model 1		Model 2	
	THI ≤ 60	THI = 78	THI ≤ 60	THI = 78
0	0.26	0.20	0.33	0.30
1	0.28	0.23	N/A	N/A
2	0.26	0.21	0.32	0.27
3	0.29	0.18	0.35	0.29
4	0.26	0.21	N/A	N/A
5	0.24	0.20	0.29	0.29
6	0.25	0.18	0.32	0.29
7	0.27	0.24	0.33	0.33
8	0.22	0.16	0.28	0.24
9	0.23	0.17	0.28	0.25
Mean (*SE*)	0.26 (0.02)	0.20 (0.03)	0.31 (0.03)	0.28 (0.03)

**TABLE 3:** Means of estimated heritabilities from 6 DIM to 305 DIM for test‐day somatic cell score in each data subset

Subset	Model 1		Model 2	
	THI ≤ 60	THI = 78	THI ≤ 60	THI = 78
0	0.06	0.08	0.07	0.08
1	0.09	0.08	0.11	0.09
2	0.09	0.09	0.11	0.10
3	0.08	0.09	0.10	0.12
4	0.10	0.11	0.10	0.10
5	0.07	0.10	0.08	0.11
6	0.07	0.08	N/A	N/A
7	0.08	0.08	0.09	0.10
8	0.10	0.11	0.10	0.13
9	0.06	0.06	0.07	0.07
Mean (*SE*)	0.08 (0.01)	0.09 (0.02)	0.09 (0.02)	0.10 (0.02)

**FIGURE 2:**
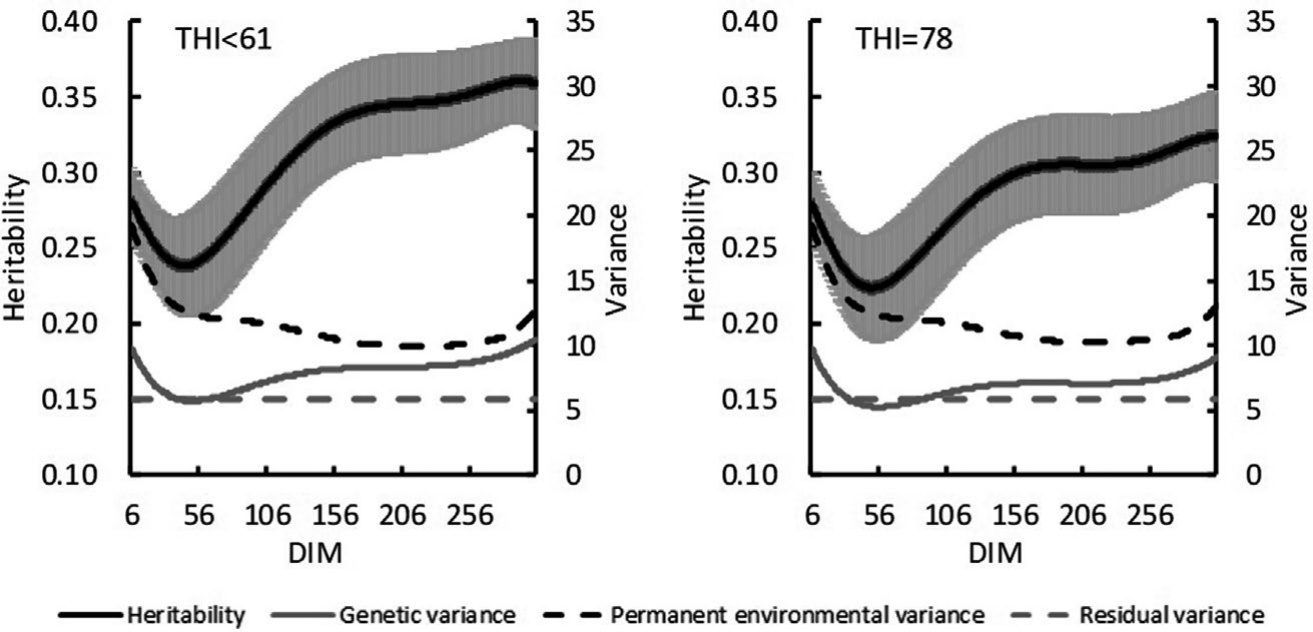
Estimates of heritabilities (and standard errors [bars]), genetic variances, permanent environmental variances, and residual variances for test‐day milk yield on days in milk (DIM) in THI ≤ 60 and THI = 78

**FIGURE 3:**
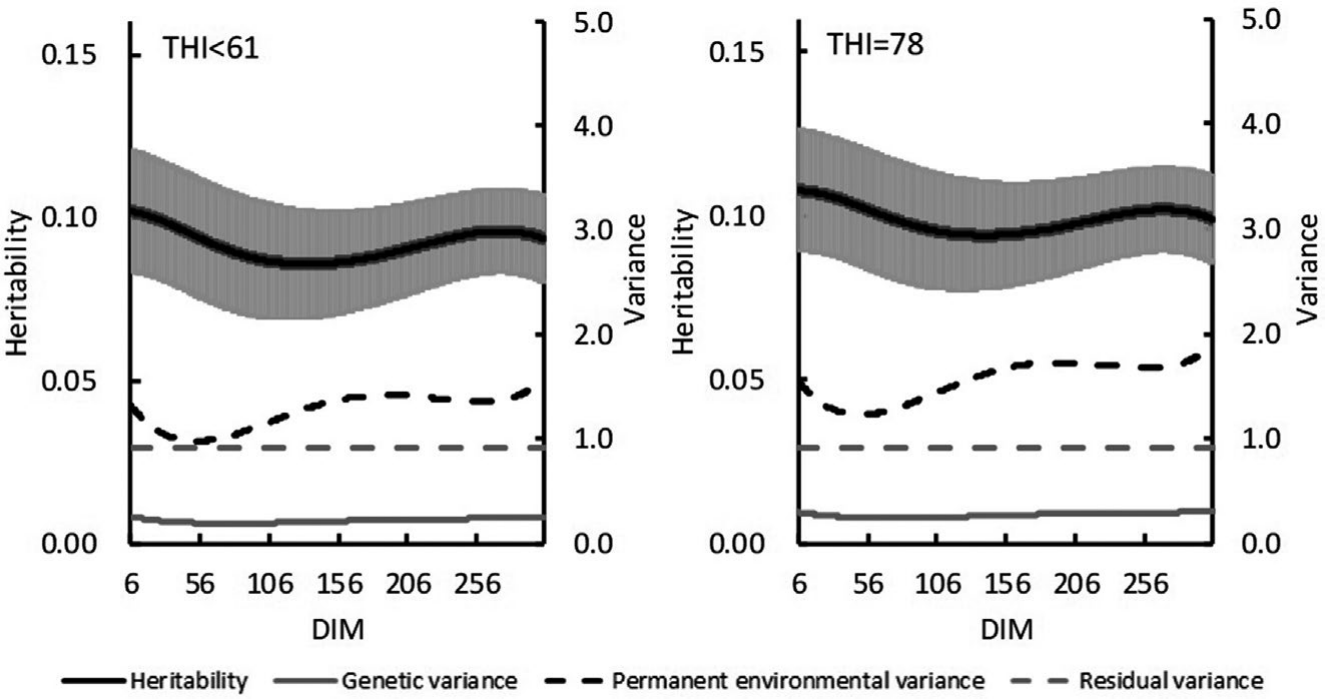
Estimates of heritabilities (and standard errors [bars]), genetic variances, permanent environmental variances, and residual variances for test‐day somatic cell score on days in milk (DIM) in THI ≤ 60 and THI = 78

In Model 2, heritability and variance estimates varied with increasing DIM. Under THI ≤ 60, estimated heritability for test‐day milk yield decreased from 0.28 at 6 DIM to 0.24 at 45 DIM and then increased to 0.36 toward the end of lactation (Figure 2). Estimated genetic variances with THI ≤ 60 ranged from 5.8 in 50 DIM to 10.5 in 305 DIM. Under the HS condition (THI = 78), estimated heritabilities first decreased from 0.28 at 6 DIM to 0.22 at 50 DIM and then increased to 0.32 as DIM increased. The mean heritability for test‐day in lactation period was 0.28, and estimated genetic variances ranged from 5.3 to 9.8. Similar trends for heritability with increasing DIM emerged for both THIs, but mean heritabilities were lower for HS (THI = 78). For SCS, heritability estimates at THI ≤ 60 (range, 0.09 to 0.10) were nearly constant among DIMs (Figure 3). Likewise, genetic variances on DIM were equivalent. Estimated PE variances on DIM were between 0.19 and 0.26. Heritability estimates under HS (THI = 78) ranged from 0.09 to 0.11. Estimated heritabilities at THI ≤ 60 paralleled those under HS conditions (THI = 78).

**FIGURE 4:**
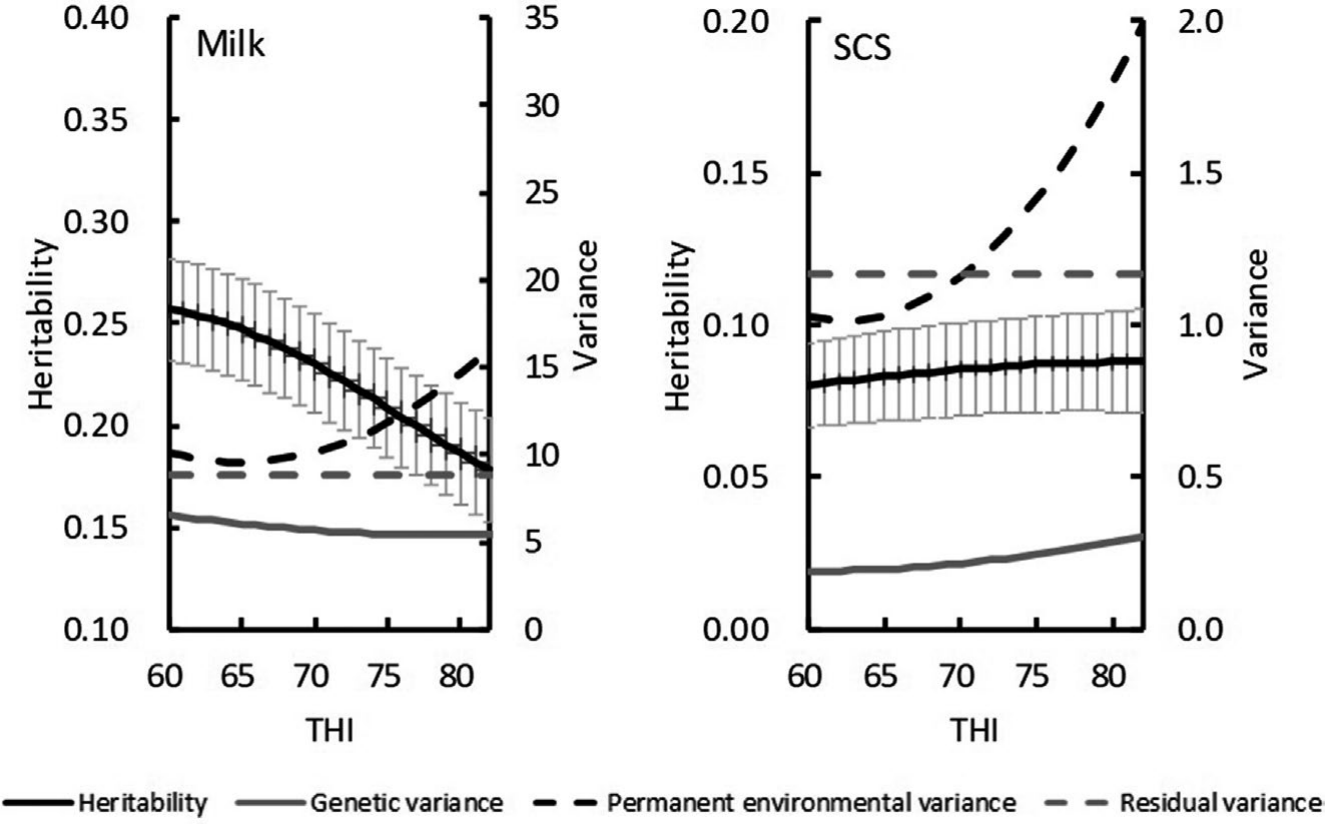
Estimates of heritabilities (and standard errors [bars]), genetic variances, permanent environmental variances, and residual variances by Model 1 for test‐day milk yield and somatic cell score (SCS) on THI

**FIGURE 5:**
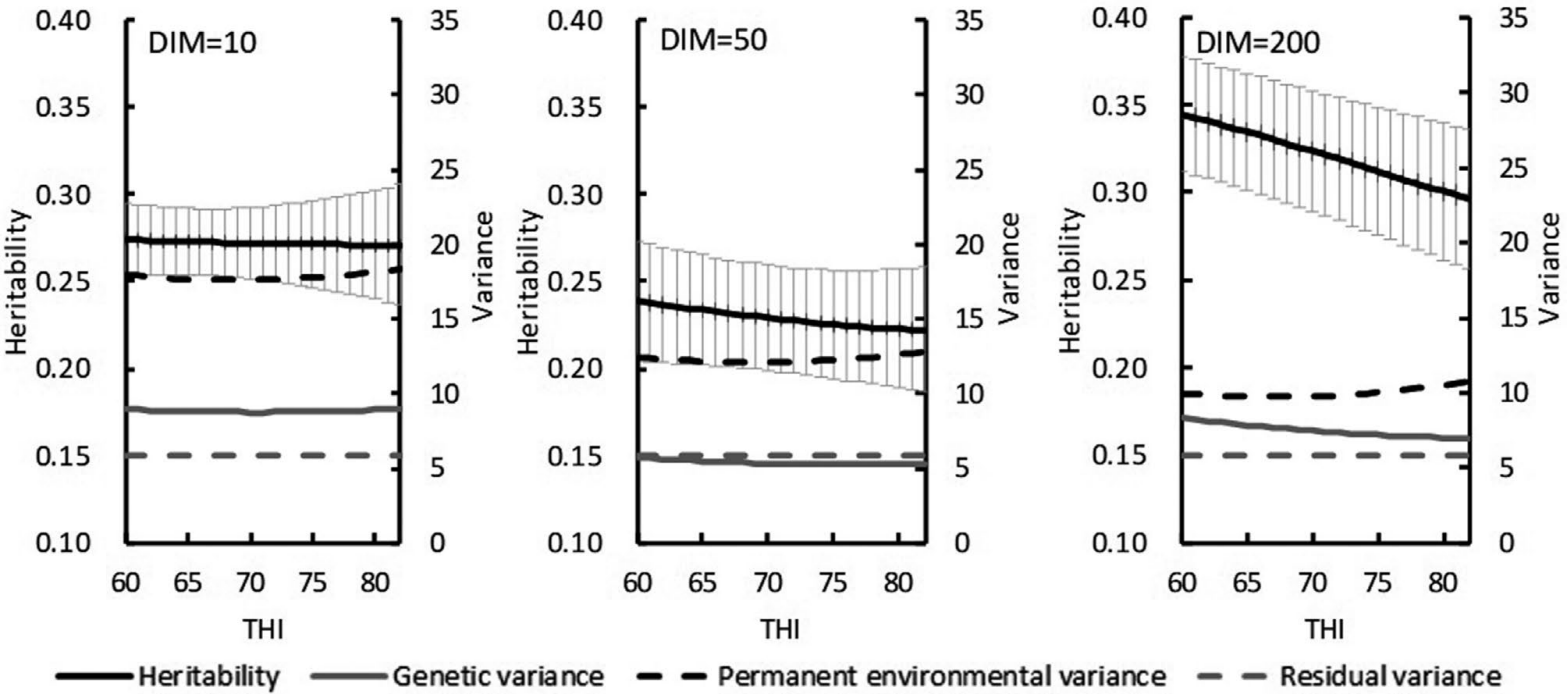
Estimates of heritabilities (and standard errors [bars]), genetic variances, permanent environmental variances, and residual variances by Model 2 for test‐day milk yield on THI at 10, 50, and 200 days in milk (DIM)

**FIGURE 6:**
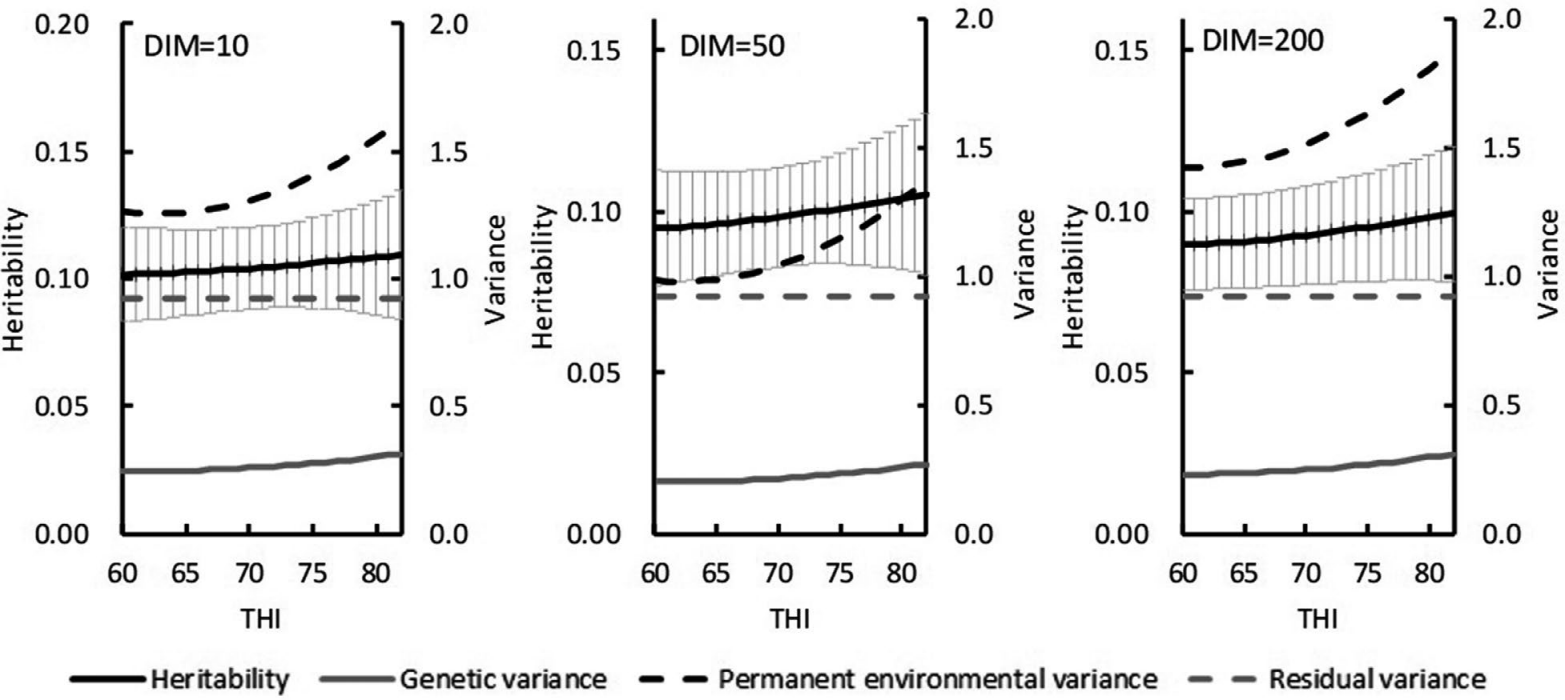
Estimates of heritabilities (and standard errors [bars]), genetic variances, permanent environmental variances, and residual variances by model 2 for test‐day somatic cell score on THI at 10, 50, and 200 days in milk (DIM)

When the values of THI exceeded a threshold (THI = 60), heritability estimates and genetic variances from Model 1 decreased with increasing THI for test‐day milk yield but did not differ for SCS (Figure 4). For both traits, PE variances increased with increasing THI. Heritability estimates for milk yield were nearly constant at 10 DIM (Figure 5) but tended to decrease with increasing THI (DIM = 200). For SCS, heritability estimates and genetic variances did not vary with increasing DIM (Figure 6).

### 3.3 Genetic correlations

**FIGURE 7:**
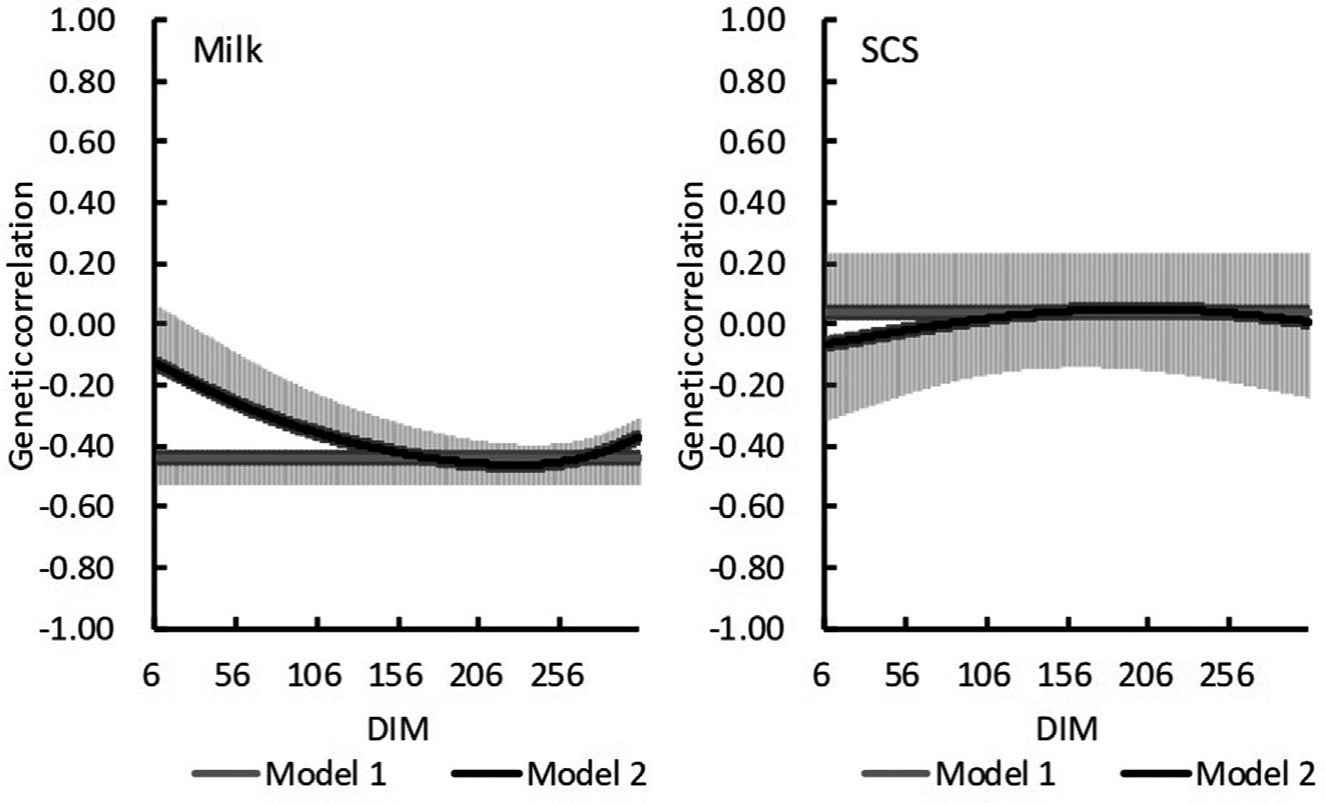
Estimated genetic correlations between general traits (milk or somatic cell score [SCS]) and heat tolerance (and standard errors [bars]) on days in milk (DIM)

Estimated genetic correlations between general and HT additive genetic effects for milk yield were –0.44 according to Model 1 and ranged from –0.13 to –0.46 with Model 2 (Figure 7). In Model 2, trends for genetic correlations between general and HT additive genetic effects on DIM for milk yield decreased with increasing DIM. In early lactation, estimated genetic correlations for milk yield tended to be weaker for Model 2 than Model 1, but estimates from both models were similar during mid‐lactation. Estimated genetic correlations for SCS were close to zero throughout lactation in both models.

### 3.4 Correlations between EBVs for heat durability

Estimated EBV showed a high correlation (0.77) between models within each milk yield and SCS class (Table 4). Correlation between models for EBVs for milk yield and SCS were negative but close to zero (range, –0.01 to –0.18).

**TABLE 4:** Correlations between estimated breeding values of heat tolerance for bulls with more than 15 daughters

	Model 1	Model 2	
	Somatic cell score	Milk	Somatic cell score
Model 1			
Milk	–0.12**	0.77**	–0.18**
SCS		–0.01	0.77**
Model 2			
Milk			–0.13**

** *p* < .01.

### 3.5 Genetic trends of heat durability

**FIGURE 8:**
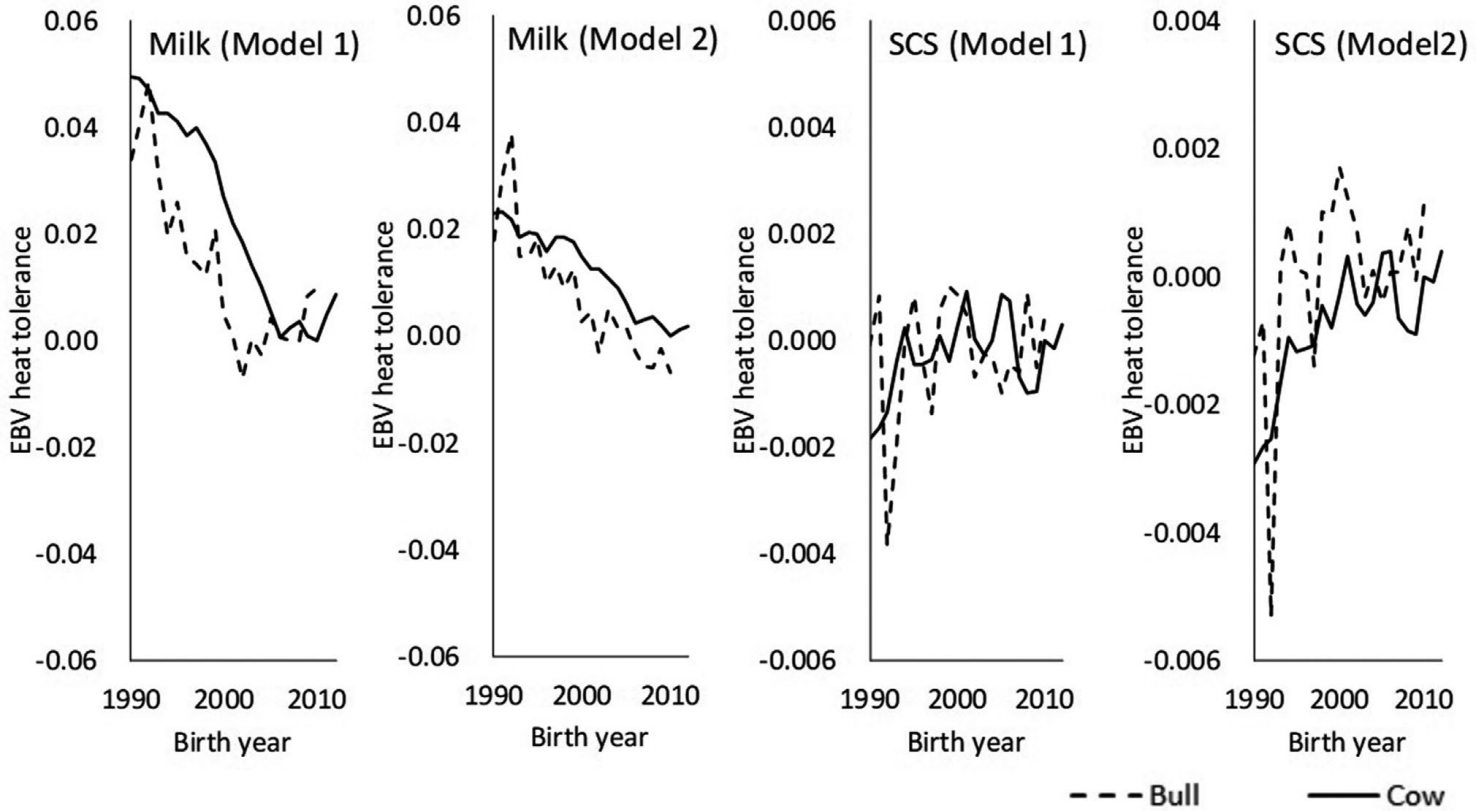
Genetic trends of heat tolerance (milk or somatic cell score [SCS]) estimated by using Model 1 or Model 2. Genetic bases are the mean EBV of cows born in 2010

Genetic trends of HT estimated by using milk yield decreased over time in both models (Figure 8). Those estimated by using SCS increased with increasing year. According to estimates from both traits, EBVs of HT in Holstein cows in Japan are changing in an undesirable direction.

## 4 DISCUSSION

Daily milk yield (range, 26.9 to 27.2 kg) and SCS (range, 2.32 to 2.34) in the current study were similar to those recently reported (daily milk yield, 26.9 kg; SCS, 2.3 to 2.5) for Holstein cows in Japan (Hagiya et al., 2017, 2019; Yamazaki et al., 2016). The shapes of the histograms (Figure 1) were almost the same as those in previous work (Hagiya et al., 2019). Approximately 30% of total days were THI > 60: that is, Holstein cows in Japan are under HS for 120 days each year.

We were unable to estimate genetic parameters for some subsets (Tables 2 and 3). When initial values for a subset were appropriate, estimation involved fewer rounds of calculation than when initial values were suboptimal. That is, when initial values were poor, estimation required numerous rounds of calculation and excessive computational time. Moreover, in some cases, we were unable to find any adequate initial values at all and therefore could not obtain appropriate estimates.

For both models, estimated heritability for milk yield was higher in the absence of HS (THI ≤ 60) than under HS conditions (THI = 78; Figure 2). In contrast, heritability estimates for SCS were similar between the 2 THI conditions (Figure 3). Aguilar et al. (2009) reported that heritability estimates for milk yields were lower in early lactation but increased with DIM and THI; heritability estimates in our study were similar to these previous estimates regarding trends on increasing DIM. Although the previous (Aguilar et al., 2009) and our current studies showed somewhat different trends regarding estimates on increasing THI, heritabilities below the HS threshold differed negligibly from those during HS. In this regard, Bernabucci et al. (2014) reported that heritability for milk yield was 0.23 without HS but 0.22 under HS conditions. Using data from southern Japan with a random regression model, Atagi et al. (2018) estimated mean heritabilities across lactation as 0.24 under THI = 60 and 0.17 under THI = 80 for milk yield and 0.06 under THI = 60 and 0.07 under THI = 80 for SCS. Our results agree with theirs for milk yield and are slightly higher for SCS, but the trends for estimates without and with HS were similar (Figure 4). In addition, our heritability estimates were constant with increasing THI during early lactation and decreased with increasing THI later during lactation (Figure 5). Although these changes were not statistically significant, they suggest that HS might have an increased effect during late lactation. Heritability estimates for SCS did not differ throughout lactation. Estimated PE variances differed between Model 1 and Model 2. Specifically, PE variances increased markedly with increasing THI in Model 1 (Figure 4), only minimally in Model 2 (Figure 5). Estimated genetic correlations both increased and decreased with increasing DIM in Model 2 (Figure 7) but remained consistent, according to the restriction applied, in Model 1. Unexplained changes in genetic variances with increasing THI in Model1 might be reflected as PE variances in Model 2.

For both of our models, estimated genetic correlations for milk yield were negative throughout the lactation period. Therefore, selection toward increasing milk yields would decrease HT in Holstein cows. In Thai crossbred cattle, estimated genetic correlations between general additive genetic and HT additive genetic ranged from –0.21 to –0.33 (Boonkum et al., 2011). Bernabucci et al. (2014) reported a moderate negative genetic correlation (–0.51) between general and HT additive genetic effects from first lactation records for Holstein cows in Italy. Atagi et al. (2018) reported negative correlations between test‐day milk yield and HT in data from southern Japan. Our results are in agreement with all of those estimates. These results indicate that selecting for increased production would decrease HT. Nguyen et al. (2017) reported that genetic trends of HT based on yield traits of Holsteins in Australia declined from 1990 to 2011. In addition, Aguilar, Misztal, and Tsuruta (2010) reported genetic trends that were close to zero in first lactation and became negative in later lactations. Because genetic trends have similarly declined recently in Japan (Figure 8), we recommend selecting bulls with high HT EBV (i.e., include EBV of HT in a selection index), owing to the decreasing correlation response with genetic improvement of milk yield. For cows born between 1990 and 2010, the decrease in mean EBV of HT for each THI unit reduces daily milk yield by approximately 0.05 kg, which is equivalent to a decrease of 15 kg per 305‐day milk yield. In our study, we estimated HT EBVs by using milk yield and SCS as indicators of HT. In practice, the 15‐kg decrease in milk yield might have a negligible effect on the net income of dairy farms. However, HS adversely affects not only milk yield and SCS but also milk fat, milk protein, female fertility, and other traits (e.g., Atagi et al., 2018; Hagiya et al., 2017). Therefore, genetic improvement of HT is very important, especially in southern Japan.

The correlation between EBV of HT was 0.77 for each trait and in both models, thus yielding similar EBVs (Table 4). However, correlations between EBVs of HT based on milk yield and on SCS were both close to zero. This finding suggests that HT EBVs estimated by using milk yield and those estimated by using SCS reflected different traits. EBV of HT with milk yield indicates HT effects on production performance, and that with SCS indicates HT effects on cow health. Nguyen et al. (2017) showed an example of EBV HT that used weighted EBVs of HT based on milk, fat, and protein. For calculating the total EBV of HT, we recommend weighting EBVs of HT based on SCS as a health indicator and EBVs of HT as production indicators based on yield traits.

## 5 CONCLUSION

We investigated genetic parameters in the responses of milk yield and SCS to HS in cows by using random regression models that incorporated daily weather records from provincial weather stations throughout Japan. In addition, we compared the EBVs of HT between estimates based on milk with those for SCS. Mean estimated heritabilities were lower during HS than when below the HS threshold for milk yield but were similar regardless of THI for SCS. Moderate negative genetic correlations between general additive genetic effects and those for HT were estimated for milk yield, but SCS showed no such genetic correlations. Correlations between EBVs of HT based on milk yield and those based on SCS were negative but close to zero.

## References

[asj13437-bib-0001] Aguilar, I. , Misztal, I. , & Tsuruta, S. (2009). Genetic components of heat stress for dairy cattle with multiple lactation. Journal of Dairy Science, 92, 5702–5711. 19841230 10.3168/jds.2008-1928

[asj13437-bib-0002] Aguilar, I. , Misztal, I. , & Tsuruta, S. (2010). Genetic trends of milk yield under heat stress for US Holsteins. Journal of Dairy Science, 93, 1754–1758. 20338455 10.3168/jds.2009-2756

[asj13437-bib-0003] Ali, A. K. A. , & Shook, G. E. (1980). An optimum transformation for somatic cell concentration in milk. Journal of Dairy Science, 63, 487–490. 10.3168/jds.S0022-0302(80)82959-6

[asj13437-bib-0004] Ammer, S. , Lambertz, C. , & Gauly, M. (2016). Is reticular temperature a useful indicator of heat stress in dairy cattle? Journal of Dairy Science, 99, 1–10. 10.3168/jds.2016-11282 27665136

[asj13437-bib-0005] Atagi, Y. , Morota, G. , Onogi, A. , Osawa, T. , Yasumori, T. , Adachi, K. , … Iwata, H. (2019). Consideration of heat stress in multiple lactation test–day models for dairy production traits. Interbull Bulletin, 55 Retrieved from https://journal.interbull.org/index.php/ib/article/view/1467

[asj13437-bib-0006] Atagi, Y. , Onogi, A. , Osawa, T. , Yasumori, T. , Adachi, K. , Yamaguchi, S. , … Iwata, H. (2018). Effect of heat stress on production traits of Holstein cattle in Japan: Parameter estimation using test day records of first parity and genome wide markers. Interbull Bulletin, 53, 9–16.

[asj13437-bib-0007] Bernabucci, U. , Biffani, S. , Buggiotti, L. , Vitali, A. , Lacetera, N. , & Nardona, A. (2014). The effects of heat stress in Italian Holstein dairy cattle. Journal of Dairy Science, 97, 471–486. 10.3168/jds.2013-6611 24210494

[asj13437-bib-0008] Bohmanova, J. , Misztal, I. , Tsuruta, S. , Norman, H. D. , & Lawlor, T. J. (2008). Genotype by environment interaction due to heat stress. Journal of Dairy Science, 91, 840–846. 18218772 10.3168/jds.2006-142

[asj13437-bib-0009] Boonkum, W. , Misztal, I. , Duangjinda, M. , Pattarajinda, V. , Tumwasorn, S. , & Sanpote, J. (2011). Cenetic effects of heat stress on milk yield of Thai Holstein crossbreds. Journal of Dairy Science, 94, 487–492. 21183060 10.3168/jds.2010-3421

[asj13437-bib-0010] Carabaño, M. J. , Bachagha, K. , Romón, M. , & Díaz, C. (2014). Modeling heat stress on Holstein cows under hot and dry conditions: Selection tools. Journal of Dairy Science, 97, 7889–7904. 25262182 10.3168/jds.2014-8023

[asj13437-bib-0011] Hagiya, K. (2019). Development of genetic evaluation for milk production traits of Holsteins in Japan. Animal Science Journal, 90, 457–461. 10.1111/asj.13190 30763985 PMC6594172

[asj13437-bib-0012] Hagiya, K. , Bamba, I. , Osawa, T. , Atagi, Y. , Takusari, N. , Itoh, F. , & Yamasaki, T. (2019). Length of lags in responses of milk yield and somatic cell score on test day to heat stress in Holsteins. Animal Science Journal, 90, 613–618. 10.1111/asj.13186 30815937 PMC6593864

[asj13437-bib-0013] Hagiya, K. , Hayasaka, K. , Yamasaki, T. , Shirai, T. , Osawa, T. , Terawaki, Y. , … Suzuki, M. (2017). Effects of heat stress on production, somatic cell score and conception rate in Holsteins. Animal Science Journal, 88, 3–10. 10.1111/asj.12617 27113198

[asj13437-bib-0014] Hammami, H. , Bormann, J. , M’hamdi, N. , Montalda, M. M. , & Gengler, N. (2013). Evaluation of heat stress effects on production traits and somatic cell score of Holsteins in temperate environment. Journal of Dairy Science, 96, 1844–1855. 23313002 10.3168/jds.2012-5947

[asj13437-bib-0015] Hayes, B. J. , Carrick, M. , Bowman, P. , & Goddard, M. E. (2003). Genotype x environment interaction for milk production of daughters of Australian dairy sires from test‐day records. Journal of Dairy Science, 86, 3736–3744. 14672205 10.3168/jds.S0022-0302(03)73980-0

[asj13437-bib-0016] Lambertz, C. , Sanker, C. , & Gauly, M. (2014). Climatic effects on milk production traits and somatic cell score in lactating Holstein‐Friesian cows in different housing systems. Journal of Dairy Science, 97, 319–329. 10.3168/jds.2013-7217 24239072

[asj13437-bib-0017] Ministry of the Environment, Ministry of Education, Culture, Sports, Science and Technology, Ministry of Agriculture, Forestry and Fisheries, Ministry of Land, Infrastructure, Transport and Tourism, Japan Meteorological Agency . (2018). Climate change in Japan and its impacts. Retrieved from https://www.env.go.jp/earth/tekiou/pamph2018_full_Eng.pdf

[asj13437-bib-0018] Misztal, I. , Tsuruta, S. , Strabel, T. , Auvray, B. , Druet, T. , & Lee, D. H. (2002). BLUPF90 and related programs (BGF90). Proceedings of the 7th World Congress on Genetic Applied to Livestock Production, CD–ROM Communication no. 28, 07, Montpellier, France.

[asj13437-bib-0019] Nagamine, Y. , & Sasaki, O. (2008). Effect of environmental factors on fertility of Holstein‐Friesian cattle in Japan. Livestock Science, 115, 89–93. 10.1016/j.livsci.2008.01.023

[asj13437-bib-0020] National Institute for Agro‐Environmental Sciences . (2017). MeteoCrop DB. Retrieved from https://meteocrop.dc.affrc.go.jp/top.php

[asj13437-bib-0021] Nguyen, T. T. T. , Bowman, P. , Haile–Mariam, M. , Nieuwho, G. J. , Hayes, B. J. , & Pryce, J. E. (2017). Implementation of breeding value for heat tolerance in Australian dairy cattle. Journal of Dairy Science, 100, 7362–7367. 28711268 10.3168/jds.2017-12898

[asj13437-bib-0022] Nguyen, T. T. T. , Bowman, P. , Haile–Mariam, M. , Pryce, J. E. , & Hayes, B. J. (2016). Genomic selection for heat tolerance in Australian dairy cattle. Journal of Dairy Science, 99, 2849–2862. 27037467 10.3168/jds.2015-9685

[asj13437-bib-0023] NRC (1971). A guide to environmental research on animals. Washington, DC: National Academy of Science.

[asj13437-bib-0024] Ravagnolo, O. , & Misztal, I. (2000). Genetic component of heat stress in dairy cattle, parameter estimation. Journal of Dairy Science, 83, 2126–2130. 10.3168/jds.S0022-0302(00)75095-8 11003247

[asj13437-bib-0025] St‐Pierre, N. R. , Cobanov, B. , & Schnitkey, G. (2003). Economic losses from heat stress by US livestock industries. Journal of Dairy Science, 86, E52–E77. 10.3168/jds.S0022-0302(03)74040-5

[asj13437-bib-0026] West, J. W. (2003). Effects of heat–stress on production in dairy cattle. Journal of Dairy Science, 86, 2131–2144. 10.3168/jds.S0022-0302(03)73803-X 12836950

[asj13437-bib-0027] Wilmink, J. B. M. (1987). Adjustment of test‐day milk, fat and protein yield for age, season and stage of lactation. Livestock Production Science, 16, 335–348. 10.1016/0301-6226(87)90003-0

[asj13437-bib-0028] Yamazaki, T. , Hagiya, K. , Takeda, H. , Osawa, T. , Yamaguchi, S. , & Nagamine, Y. (2016). Effects of stage of pregnancy on variance components, daily milk yields and 305–day milk yield in Holstein cows, as estimated by using a test‐day model. Animal, 10, 1263–1270. 10.1017/S1751731116000185 26906742

